# Cancer-Associated Thrombosis: Not All Low-Molecular-Weight Heparins Are the Same, Focus on Tinzaparin, A Narrative Review

**DOI:** 10.1155/2022/2582923

**Published:** 2022-07-19

**Authors:** Agnese Maria Fioretti, Tiziana Leopizzi, Agata Puzzovivo, Francesco Giotta, Vito Lorusso, Giovanni Luzzi, Stefano Oliva

**Affiliations:** ^1^Cardio-Oncology Unit, IRCCS Istituto Tumori “Giovanni Paolo II”, Viale Orazio Flacco 65, 70124 Bari, Italy; ^2^Cardiology-Intensive Care Unit, Ospedale SS. Annunziata, Via Francesco Bruno 1, 74121 Taranto, Italy; ^3^Medical Oncology Unit, IRCCS Istituto Tumori “Giovanni Paolo II”, Viale Orazio Flacco 65, 70124 Bari, Italy

## Abstract

Cancer-associated thrombosis (CAT) is the second main cause of cancer death with high related mortality and morbidity, leading to anticancer agent delays and interruptions. The recommended therapy, low-molecular-weight heparin (LMWH), however, is burdensome for patients and costly for society, as treatment should last until cancer is no longer active, even indefinitely. Tinzaparin is a manageable, efficient, safe, and cost-effective option. Compared to the other LMWHs, advantages are single-daily dose and safety in the elderly and those with renal impairment (RI). The purpose of this review is to critically discuss recent data on its efficacy and safety in CAT.

## 1. Introduction

**Figure 1:**
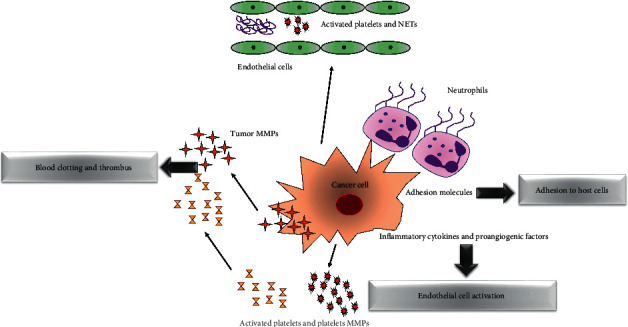
Mechanisms and pathogenesis of cancer-associated thrombosis.

CAT is a frequent complication in cancer, with an incidence of 20% [1]. It includes deep vein thrombosis (DVT) and pulmonary embolism (PE) [2] and is a potentially life-threatening disease [3]. A close and multifactorial two-way relationship exists between malignancy and venous thromboembolism (VTE), due to the alterations of coagulation factors, resulting in a host high prothrombotic burden. Overall, patient-related factors, cancer-related factors, treatment-related factors, and biomarkers concur to the development of a hypercoagulable state in cancer patients [4]. Cancer cells initiate procoagulant mechanisms, releasing microparticles (MMPs), and activate the procoagulant phenotype of endothelial cells, leukocytes, and platelets. Furthermore, neutrophils release neutrophil extracellular traps (NETs) to build a vasculature network, providing platelet adhesion and activation together with thrombin generation (Figure 1). On the other hand, thrombus formation results in a microenvironment contributing to tumor growth and dissemination. Indeed, the procoagulant scenario is responsible for the transformation of “dormant” cells into cells capable of malignant activities [5]. CAT is not only a major cause of morbidity and anticancer delays or discontinuations, but mostly a sign of worse prognosis. Indeed, cancer patients with CAT present a lower overall survival rate compared to cancer patients without CAT [6]. In particular, the risk of CAT varies according to cancer sites and stages, pancreas, brain, lung, ovarian, and multiple myeloma being the sites related to the highest VTE risks. In addition, locally advanced and metastatic tumors present a higher VTE risk compared to localized stages [7]. Current guidelines still advocate LMWH for VTE therapy and secondary prevention for at least 6 months and for primary prophylaxis in bedridden medically ill patients, patients following major abdominal and pelvic surgery, and outpatients with VTE intermediate-high risk, without distinctions between LMWH agents [8]. However, LMWHs are not all alike due to substantial differences in their pharmacokinetic profiles and, accordingly, pharmacodynamic effects or risks in RI [9]. LMWH is a favorable choice also in cancer patients unable to take oral pills due to nausea, vomiting, or anorexia [10]. Of note, tinzaparin presents a number of advantages compared to other LMWHs, including a once-daily curative dose, no need for a reduced dose over time, and a clear clinical benefit in the elderly and those with RI, essential in cancer patients who might require anticoagulant therapy indefinitely [11]. Nevertheless, tinzaparin remains relatively unknown and underused in CAT clinical practice [12].

## 2. Methods

The purpose of this review is to provide an update regarding the therapeutic and prophylactic use of tinzaparin use in medical and surgical adult cancer patients, including special cancer populations such as those with RI. PubMed was searched up to December 2021 for original articles in English using the keywords: “tinzaparin,” “low-molecular-weight heparin,” “cancer,” “venous thromboembolism,” “deep vein thrombosis,” “pulmonary embolism,” “renal impairment” and “kidney disease”. Papers regarding haemodialysis or haemofiltration were excluded.

## 3. Pharmacological Profile

Tinzaparin is the product of unfractionated heparin (UFH) depolymerisation [13]. It enhances the inhibition effect of antithrombin III on clotting factors (Factor Xa > Factor IIa) through the highest release of all LMWHs of Tissue Factor Pathway Inhibitor (TFPI), a strong coagulation inhibitor. Anti-Xa Factor level is the biomarker for LMWH activity. Tinzaparin possesses the lowest anti-Xa/anti-IIa activity ratio among all LMWHs and, hence, the highest rates of anti-Xa reversal in response to protamine sulphate. It has the highest molecular weight of all LMWHs [14]. Tinzaparin applies first-order pharmacokinetics, mainly involving cellular and liver routes of elimination and, to a lesser degree, renal clearance (RC). There is no bioaccumulation even in severe RI, it requires no dose-adjustment and, notably, there is less risk of bleeding [15]. Its elimination half-life is 3.4 to 4.1 hours after subcutaneous injection and 1.6 hours after intravenous administration. Tinzaparin administration does not affect haemoglobin level or platelet count [16].

## 4. Evidence in Cancer-Associated Thrombosis

### 4.1. Treatment

In the LITE trial, a multicenter open-label randomized controlled trial (RCT), 200 patients with CAT were randomized to receive tinzaparin or their usual anticoagulant care (UAC), warfarin, for 3 months [17]. At the 12-month follow-up, the DVT recurrence rate was 16% in the warfarin group and 7% in the tinzaparin group (*P* = 0.44; risk ratio RR = 0.44; absolute difference AD: − 9.0; 95% confidence interval CI: − 21.7% to − 0.7%). Tinzaparin was not associated with an increased overall bleeding risk compared to warfarin (27% in tinzaparin, 24% in UAC). Mortality was 47% in each arm. Despite the small sample size, the above results are in accordance with the CLOT Study [18], a benchmark international multicenter open-label randomized trial on the role of dalteparin, which showed that LMWH is more effective than warfarin in long-term CAT therapy. In Main-LITE Trial, a multicenter open-label RCT, 737 patients with VTE (27% with cancer) were divided into 2 arms: tinzaparin therapeutic dose versus vitamin K antagonists (VKA) [19]. At 3-month follow-up, mortality and effectiveness were the same in both arms. VTE recurrence occurred in 4.9% in the tinzaparin arm and 5.7% in UAC (AD: − 0.8%, 95% CI: − 4.1 – 2.4). Overall bleedings occurred in 13% of patients in the tinzaparin group and in 19.8% with warfarin (AD: − 6.8%; *P* = 0.11; RR = 0.66). Furthermore, major bleeding events ceased early with LMWH (by day 23, *P* = 0.34), but persisted throughout the VKA study. Hence, tinzaparin was not inferior to UAC in VTE treatment and was also safer. Significantly, it allowed greater patient autonomy and recourse to fewer healthcare resources. In the Home-LITE Trial, a multicenter open-label RCT, 480 patients with acute DVT (25% had cancer) were divided into a tinzaparin arm and a warfarin arm, to compare long-term home-based treatment [20]. VTE recurrence rates were 3.3% in both groups (AD: 0%; 95% CI: − 3.2 – 3.2) during the 12-week study period and also at 1-year follow-up (10.4%/8.3% in the tinzaparin/UAC groups, respectively; difference: 2.1%; 95% CI: − 3.1 – 7.3). Mortality and bleeding rates were also similar. Perceived treatment satisfaction, assessed via a questionnaire, showed that patients in the tinzaparin arm were significantly more satisfied (*P*: 0.024), especially regarding lack of interference with daily activities. Unlike the CLOT Study, in which patients were treated at home “whenever possible” and hospitalized when required by their physician, patients in the Home-LITE Trial were all suitable for homecare [21]. The Home-LITE Trial retrospective subanalysis demonstrated tinzaparin superiority (overall odds ratio OR: 0.76, *P*: 0.004) over warfarin in significantly reducing the incidence of venous ulcers and post-thrombotic syndrome (PTS), a frequent, difficult and costly VTE sequela. The greatest benefit was found in patients with iliac vein thrombosis (OR: 0.53), frequently associated with worse prognosis. Its ability to prevent PTS may be due to the longer chain length, resulting in a greater release of TFPI, a molecule with anticoagulant, anti-inflammatory and antiangiogenetic effects [22]. The CATCH Study, a multicenter open-label RCT, is the largest (900 patients) treatment investigation in patients with CAT. Tinzaparin was administrated for 6 months at full therapeutic dose (175 UI/kg/die) [23]. Study treatment duration was longer in the tinzaparin group than in the warfarin group (168 days versus 127 days, respectively), possibly introducing a bias in favour of tinzaparin. Tinzaparin did not significantly reduce VTE recurrence and overall mortality or major bleedings, but it did reduce the risk of clinically relevant non major bleeding (CRNMB) versus warfarin (OR: 0.58, *P*: 0.04) [24]. The CATCH Study enrolled patients with symptomatic VTE (s-VTE) and incidental VTE (i-VTE), while the CLOT Study only considered s-VTE. As regards i-VTE, in a multicenter prospective observational study on 120 patients with CAT (35% with i-VTE), Papakotoulas et al. reported that tinzaparin was effective and safe for at least 6 months; only 3 patients had VTE recurrence and 4 patients minor bleedings. Moreover, i-VTE contributed significantly to CAT burden; indeed, i-VTE presents the same outcomes and mortality rates as s-VTE; thus published guidelines recommend the same treatment for both conditions [25]. The ability of tinzaparin to reduce VTE recurrence appeared to be inferior in the CATCH versus the CLOT Study, as the expected recurrence rate of VTE in the warfarin group was 12.6% while the observed rate was only 10.9%, making it difficult to detect a beneficial effect of tinzaparin in terms of efficacy. This consideration reflects patient population differences; indeed, in the CATCH Study, cancer was less aggressive than in the CLOT Study. As reported in the prespecified secondary analysis of the CATCH Study, clinically relevant bleeding (CRB) events occurred in 15.3% of patients (13.4% in tinzaparin, 17.3% in warfarin). Cumulative incidence rates of CRB in the 2 groups diverged almost immediately after the start of therapy and continued to show a benefit for tinzaparin throughout treatment. [26]. The safety of tinzaparin in CAT patients is of utmost note, given that cancer patients on anticoagulant therapy for VTE are more likely to develop both VTE recurrence and bleedings than non cancer patients [27]. The mortality rate was higher in the CATCH Study compared to the CLOT Study, probably due mostly to the 10-year time gap between the 2 trials during which anticancer drugs improved in effectiveness. If its patient population had been at higher risk for VTE, the CATCH Study may have had better findings regarding efficacy. In a prespecified analysis of the CATCH Study, elevated Tissue Factor (TF) (> 64.6 pg/mL), compression by mass or adenopathy, diagnosis of hepatobiliary malignancy, and elevated C-reactive protein levels were risk predictors for VTE recurrence. Interestingly, the strong association between high TF levels and subsequent VTE recurrence demonstrated that TF is a potential biomarker of VTE recurrence [28]. A multicenter open-label RCT was conducted on 241 patients with VTE randomized to tinzaparin (27% had cancer) or acenocoumarol (AC) (30.3% had cancer) [29]. After 6 months, in the cancer patients VTE recurrence occurred in 5.5% of patients in the tinzaparin arm and in 9.1% of those in the AC arm, while at 12 months VTE recurred in 5.5% of cancer patients in tinzaparin arm and 21.2% in AC. Of note, the VTE recurrence rate in non cancer patients was 4.2% in the tinzaparin arm and 5.7% in the AC arm at 6 months and 5% in tinzaparin arm and 9.1% in AC arm at 1 year. Therefore, tinzaparin was more effective than AC in cancer patients compared to non cancer patients. Bleedings were not significantly different between the 2 arms, with no fatal events. Furthermore, tinzaparin contributed more than AC in venous recanalization. Indeed, complete thrombus regression was achieved in 73.1% of patients in the tinzaparin arm and in 47.5% in the AC arm after 6 months and differences continued even after 12 months (91.5% versus 69.2%, respectively). A small sample size open-label multicenter RCT (102 patients, 8% had cancer) revealed in long-term treatment (6 months) of haemodynamically stable PE that the sum of VTE recurrence and bleeding events was low and not statistically different between the tinzaparin and the VKA arms (3.8% and 2%, respectively, *P*: 0.52). Notably, in the tinzaparin group there were fewer minor bleedings, although 41.2% of patients were elderly (≥ 75 years) and had moderate to severe RI. Interestingly, there were no statistical differences in the average cost of care between the 2 arms. In particular, the lack of need for laboratory monitoring in the tinzaparin arm resulted in cost savings compared to the VKA arm; indeed, tinzaparin was not more expensive than VKA [30]. Similar findings were found in a previous open-label prospective RCT that examined tinzaparin versus AC for long-term treatment (6 months) of VTE with a small sample (n: 108) and a reduced cancer representation (6 in the tinzaparin group and 8 in the AC group) [31]. Tinzaparin was at least as effective and safe as AC and, interestingly, it performed better in recanalization than AC, given that thrombus lysis appeared significantly earlier and more extensively from 3 months onwards (*P* < 0.2). These findings are particularly important considering that residual proximal DVT diagnosed by compression ultrasonography after 6 months of treatment is associated with thrombophilia [32]. Laporte et al. evaluated 5 open-label RCTs to investigate tinzaparin versus VKA for long-term treatment of VTE. In cancer patients, the meta-analysis (1668 patients, 24% had cancer) showed a 38% non significant VTE relative risk reduction (RR: 0.62, *P*: 0.21) in the tinzaparin arm at the end of 3–6-month follow-up which increased to 59% (RR: 0.41, *P*: 0.08), becoming significant at 1 year. In contrast, no difference was noted in the general population for tinzaparin use in VTE patients at any follow-up. There were no statistically significant differences in MB and mortality. Tinzaparin appeared as a valuable alternative to VKA for cancer patient therapy with a more favorable benefit-risk ratio, but only at 1-year follow-up [33]. A systematic review and meta-analysis investigated 3 RCTs (CATCH, LITE, and ROMERA, 1169 patients with cancer), comparing tinzaparin to VKA for CAT long-term treatment for at least 3 months, resulting in a risk reduction of VTE recurrence with a similar effect on all-cause mortality and overall bleedings. There was a substantial decrease in VTE at the end of treatment (RR: 0.67) and also at longest follow-up (RR: 0.58). Tinzaparin-treated patients showed lower rates of CRNMB at the end of treatment, while non significant between-group differences were found for all-cause mortality (RR: 1.09), fatal and non-fatal MB events (RR: 1.06). Quality of evidence was moderate due to the small sample sizes and the low number of events in 2 of the trials (LITE and ROMERA) and, mostly, to the fact that the largest study (CATCH) did not include 12-month follow-up. Nevertheless, strengths were the consistency and the wide range of cancer sites tested in the studies [34].

### 4.2. Secondary Prophylaxis beyond 6 Months

A retrospective cohort study involving 250 cancer patients with acute VTE treated for at least 3 months documented that early (before 6 months) cessation of anticoagulant therapy (tinzaparin or VKA) led to an 8-fold higher recurrence risk (OR: 7.2, *P*: 0.02) [35]. In the TiCAT Study, a multicenter, open-label, single arm prospective trial, on 247 patients with CAT, tinzaparin was tested for its safety and efficacy in 12 months of treatment. 80% of patients completed 6-month follow-up and 55% completed 12 months [36]. The mortality rate at 6 and 12 months was 15% and 25%, respectively. The CRB event rate was 0.9% in the first 6 months and 0.6% in a 7–12-month period (*P*: 0.5). The recurrence rate was 4.5% in the first 6 months and 1.1% in a 7–12-month period. Therefore, the overall results supported the use of tinzaparin as a safe drug for CAT extended treatment beyond 6 and up to 12 months, as the rate of VTE recurrence and MB was low. Despite the favourable findings of the TiCAT Trial, the DACUS Study evidenced that, in patients at low VTE risk for the absence of residual VTE, prolonging anticoagulation therapy did not add any benefit compared to stopping it after 6 months [37]. 2 prospective observational cohort studies documented the use of tinzaparin in CAT patients for 6 months: the Predicare Study [38] and the aXa Study (NCT02898051; https://clinicaltrials.gov/ct2/show/NCT02898051). Subsequently, 432 patients enrolled in these 2 studies were available to participate in the USCAT Study, a retrospective non interventional study, to describe long-term follow-up in CAT therapy with tinzaparin from the 6^th^ to the 12^th^ month following the index VTE [39]. Between 6 and 12 months, 5.7% of patients experienced VTE recurrence, while 5.1% had CRB, 2.7% had MB, and 22.3% died, mostly from cancer. VTE recurrence was more frequent in lung (14.3%) and colorectal cancer (6.0%), while MB was more frequent in colorectal cancer (6.0%). The study identified the cancer types particularly associated with VTE recurrence or bleeding and suggested that, in the absence of robust data from RCTs, it is likely to provide useful guidance for the long-term use of tinzaparin in CAT. A recent abstract report on a subgroup analysis of the prospective observational TROPIQUE Study [40] documented the long-term use of tinzaparin in 301 patients with CAT. Mean tinzaparin treatment duration was 5.0 ± 1.9 months. Over 6-month follow-up, the overall incidence of recurrent VTE was 6.0%, while the overall incidence of MB was 6.6%. Recurrent VTE (9.3%) and MB (9.3%) were more frequent in lung cancer patients compared to other cancer sites, although not statistically significant. A high incidence of MB was observed also in hematopoietic cancer patients (9.8%). These findings confirm a favourable benefit-risk ratio of tinzaparin for CAT long-term use and that clinical outcomes may differ according to cancer site [41].

### 4.3. Primary Surgical Prophylaxis

In a single-arm open-label pilot trial, tinzaparin (4500 IU/die) was used in 40 patients with grade III-IV malignant glioma undergoing intracranial surgery to evaluate safety for primary prophylaxis [42]. 5% of patients developed central nervous system (CNS) haemorrhage (grade 1–2), which was acceptable and comparable to CNS bleeding rates reported in the CLOT Study (7%) and only 1 patient suffered from VTE while receiving tinzaparin. Therefore, tinzaparin was safe and able to decrease VTE incidence in brain tumors. In a Danish national registry study, 8645 patients (4273 without cancer and 4372 with cancer) underwent major renal surgery and 2164 patients (359 without cancer and 1805 with cancer) underwent cystectomy. Tinzaparin was used for primary postsurgical prophylaxis. After 6 months, there was no difference in VTE event rate (0.4% and 0.3%; *P*: 0.91) both in major renal surgery and in cystectomy (1.3% and 0.8%; *P*: 0.44). No VTE-related death was recorded. Furthermore, there was a significant economic advantage in the use of tinzaparin; the estimated cost for 28 days of self-injected tinzaparin at prophylactic dose was €112, while the cost if administered by a nurse was €1.988, a clear cost benefit [43]. In a retrospective cohort study, 643 patients, who underwent gynaecological cancer surgery before current guidelines, received prophylaxis with tinzaparin only during the hospital stay and were compared with a 740-patient cohort who received tinzaparin prophylaxis also for up to 4 weeks after surgery. There were no differences between the 2 prophylactic strategies, neither for thrombosis-free survival at 1-year follow-up nor for VTE recurrence rates [44]. In a prospective cohort study involving 76 patients with colon cancer, a prophylactic tinzaparin dose administrated after surgery normalized Vascular Endothelial Growth Factor (VEGF) values, whose postoperative increase is responsible for enhancing tumor growth and metastases formation due to surgical trauma-induced platelet activation. Despite the small sample size, these findings highlight the pleiotropic properties of tinzaparin, in addition to its antithrombotic ability [45]. Indeed, although it is still in infancy, its antimetastatic [46], antiangiogenetic, antidyslipidemic, and anti-inflammatory effects offer a new scenario for non traditional indications [47]. As regards cancer survival, LMWH benefits could be due not to the antithrombotic effect, but to the direct antitumoral effect and to the antiangiogenetic and immunomodulatory properties. Findings on the antitumoral effects of LMWH are mostly based on studies on cell lines and mice models [48, 49]. A randomized single-blind multicenter phase II clinical trial enrolled 100 esophageal cancer patients assigned to the chemoradiotherapy-only arm or to the chemoradiotherapy plus enoxaparin arm. Results showed a non significant trend toward improved survival by adding enoxaparin to the concurrent chemoradiotherapy treatments [50]. Moreover, although the population was relatively homogenous for disease type and histology, it was limited for the small sample size. In addition, PaCT (Pancreatic Cancer and Tinzaparin) is a retrospective observational study conducted in 110 patients with advanced or metastatic pancreatic cancer with the aim of examining the anticancer effect of tinzaparin. The patients receiving “hyper-prophylactic” dose of tinzaparin (10000 anti-Xa IU/die) during chemotherapy with nab-paclitaxel and gemcitabine had 39.5% higher progression-free survival compared to patients without tinzaparin (*P* < 0.05) [51]. However, overall, evidence on the antitumoral activity of LMWH is still contradictory and not conclusive. Accordingly, none of the current guidelines endorse LMWH with the aim of improving survival of cancer patients and this indication is off label [52].

## 5. Renal Insufficiency

LMWHs less dependent on renal clearance, such as tinzaparin, may be preferred in patient populations with a high prevalence of RI, in particular the elderly and cancer patients [53]. Indeed, compared to the other LMWHs, tinzaparin tends to bioaccumulate less in patients with RI when repeated prophylactic or therapeutic doses are administered [54]. The IRIS Study was a challenging international multicenter interventional open parallel-group RCT, since it regarded 539 elderly patients with DVT and moderate-to-severe RI, usually excluded from clinical studies. It was prematurely stopped due to a difference in mortality favouring the UFH group (4.8% had cancer) versus the tinzaparin group (7.5% had cancer) (6.3% vs. 11.5%, *P*: 0.35) [55]. Nevertheless, a post hoc multiple regression analysis showed that baseline comorbidities were statistically significant related to death, comprising the ongoing malignancy (*P* < 0.01). When the results were adjusted for baseline risk factors, mortality was no longer correlated with the treatment group. The IRIS substudy, conducted in 87 patients from only the tinzaparin group (10.3% had cancer, mean age 83 years, mean CrCl 41 ml/min), detected no accumulation of anti-Xa activity and there was no need for its systemic laboratory monitoring. No difference between patients with and without cancer was found (*P*: 0.82) [56]. Given that excessive peak anti-Xa is accepted as associated with increased bleeding risk, these findings confirm the safety profile of tinzaparin in fragile cancer categories such as the elderly and those with RI. In a secondary analysis of the CATCH Study, 15% of patients had RI (GFR < 60 ml/min) at baseline and an additional 21% experienced RI during follow-up. The recurrent VTE rate was 14% in patients with RI and 8% without RI (RR: 1.74) and CRB occurred in 19% of patients with RI versus 14% without RI (RR: 1.33). MB was 6.1% and 2% (RR: 2.98), respectively, with no statistically significant difference between the tinzaparin and the warfarin arms in each renal group. Even patients with GFR ≤ 20 ml/min received full therapeutic dose tinzaparin without any adjustment. The mortality rate in patients with RI was 40.3% compared to 33.7% in those without RI (RR: 1.2). Therefore, full therapeutic doses of tinzaparin without adjustment in CAT long-term treatment do not increase VTE recurrence rate, MB, CRB, and mortality [57]. Accordingly, treatment doses do not require monitoring or adjustment in patients with CrCl ≥ 20 ml/min. Cancer and the Kidney International Network recommended tinzaparin as the treatment of choice in cancer patients with chronic RI [58]. A systematic review regarding tinzaparin safety, including a total of 1588 cancer patients with RI, stated that MB were between 0.8% and 7% and CRNM were significantly lower compared to VKA. These results confirm tinzaparin as a safe choice. Periodic therapeutic or prophylactic doses did not result in bioaccumulation, even in severe RI [59].

## 6. Conclusions

VTE is a frequent and potentially fatal complication of cancer. LMWHs still remain the mainstay of treatment, despite concerns raised about safety and manageable use. They are regarded as interchangeable, despite being distinct pharmacological agents. Tinzaparin offers particularly convenient benefits: once-daily administration, safety in the elderly, and no need for dose adjustment in RI which likely leads to underdosing with increased thrombosis risk. For all the aforementioned advantages, tinzaparin appears primarily useful in long-term and, even, extended treatment, as requested if cancer persists. However, evidence is limited on tinzaparin's role in CAT. From a total of 28 reviewed papers, we found only 2 RCTs designed exclusively for cancer patients and 5 RCTs that studied cancer patients as a subpopulation (overall, less than 27% had cancer). No RCTs were available in patients receiving tinzaparin for CAT primary prophylaxis. Most of the studies included in this review lacked a control arm and were not large enough except for the CATCH Study. To better provide reliable results on the use of tinzaparin in the context of CAT, further RCTs conducted ad hoc in cancer patients are required. Head-to-head studies comparing tinzaparin to the other LMWHs in malignancy are warranted and, notably, aiming to adequately examine severe RI with CrCl ≤ 20 ml/min.

## Data Availability

Data are available on request (corrisponding author: Agnese Maria Fioretti, a.fioretti@oncologico.bari.it).

## Additional Points

*Key Points.* (i) CAT is an insidious complication of cancer; 1 out of 5 patients develops VTE during the clinical course of the disease, developing increased medical burden. CAT is a physically and emotionally distressing phenomenon and, notably, it is closely correlated to poor prognosis. (ii) Although currently direct oral anticoagulants are a priority over LMWHs, the latter still remain the endorsed treatment for CAT and are the necessary choice in special clinical settings such as thrombocytopenia, RI, gastrointestinal cancer, brain tumors, catheter-related thrombosis, body weight extremes, drug-drug interactions, and recurrent VTE during anticoagulation. (iii) However, the majority of LMWHs are very impractical, as they require twice-daily therapeutic self-injections with harmful effects on costs and quality of life. On the other hand, tinzaparin requires a single-daily therapeutic administration and its pharmacokinetic features make it a well-tolerated and valuable option, particularly in the elderly and patients with RI.
